# Misdiagnosis of abdominal aortic dissection: image findings with the Nellix endovascular aneurysm sealing system

**DOI:** 10.1259/bjrcr.20150318

**Published:** 2016-05-19

**Authors:** Peter James de Souza, Alexander Rodway, Ajay Pankhania

**Affiliations:** ^1^ East Surrey Hospital, Redhill, UK; ^2^ Department of Vascular Surgery, East Surrey Hospital, Redhill, UK; ^3^ Department of Radiology, East Surrey Hospital, Redhill, UK

## Abstract

The Nellix endovascular aneurysm sealing system is a relatively new aortic endoprosthesis designed to overcome the problems associated with traditional methods of endovascular aneurysm repair. We report a case in which a 65-year-old male with abdominal pain was mistakenly diagnosed with an acute aortic dissection 7 days postoperatively after Nellix stent insertion, on the basis of the CT angiography findings. This report highlights the typical radiological appearances of the Nellix stent.

## Clinical presentation

**Figure 1.**
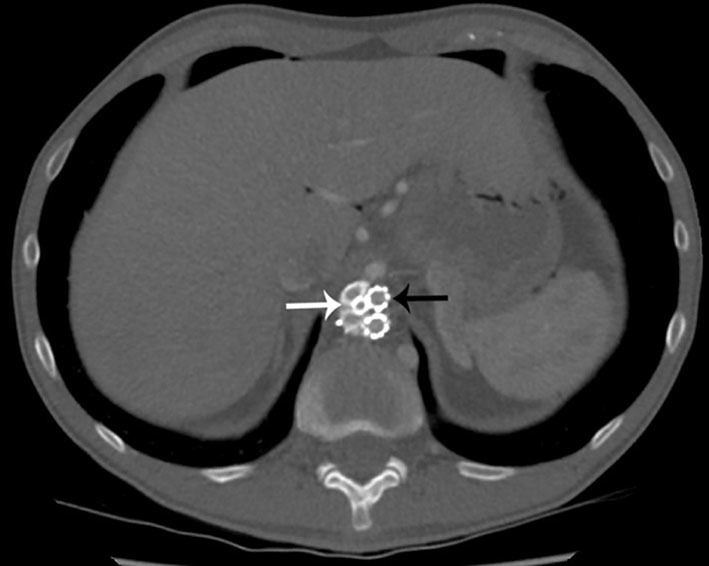
Axial CT image demonstrating the three-vessel chimney graft (black arrow) and the Nellix endograft (white arrow) within the abdominal aorta.

A 65-year-old male presented with an abdominal aortic aneurysm (AAA) that was identified on plain film imaging. He was referred to the vascular clinic for review where a 6-month history of abdominal swelling was elicited, in association with a non-tender, pulsatile abdominal mass. Subsequent abdominal ultrasound scan revealed an AAA. A CT angiogram (CTA) of the aorta demonstrated a juxtarenal AAA with a maximum diameter of 7.8 × 7.0 cm, with no evidence of leak, rupture or dissection. He was then admitted for an elective repair in which a three-vessel chimney graft, with covered stents (Atrium Medical Corp/Maquet Cardiovascular, Hudson, NH) was inserted into the superior mesenteric artery and both renal arteries, and a Nellix aortic endograft (Endologix, Inc., Irvine, CA) was inserted into the abdominal aorta (Figure 1). Post-operative endograft appearances were satisfactory, with patent visceral vessels and no evidence of endoleak. There were no post-operative complications, and the patient was discharged 3 days later.

The three-vessel chimney graft technique involves using balloon-expandable covered stents that are inserted parallel to the aortic endograft to maintain patency of the target vessels and ensure adequate end-organ perfusion when used in conjunction with the aortic stent graft, which may otherwise occlude the visceral vessels. The use of the stents in this way remains off the manufacturer’s official "instructions for use", but the technique is reported in the literature and often used in cases with unfavourable proximal aneurysm neck anatomy and where other techniques are also not feasible.^1^

A further 3 days following discharge, the patient presented to the emergency department with severe lower abdominal pain, associated with constipation (bowels had not been open for 7 days since the surgery). The pain was described as a severe sharp pain radiating to the chest. In addition to the recent stent graft insertion, the past medical history was significant only for a recent aortic valve repair. Examination revealed an abdomen that was tender in the suprapubic region with minimal bowel sounds. Observations were otherwise stable and the patient was afebrile.

## Differential diagnoses

Differential diagnosis of abdominal pain in the intermediate post-operative period includes aneurysm rupture, either owing to delayed identification of perioperative damage to the sac or as a result of a major endoleak (the persistence of arterial flow in the aneurysm sac despite the repair).^2^ Endoleak is the most common complication following endovascular aneurysm repair (EVAR),^3^ although it is usually asymptomatic. Other possibilities include ischaemic colitis, mesenteric ischaemia and stent graft infection. Unusual causes of lower abdominal pain can include wound complications in the groin, as well as injury to the access vessels, resulting in pseudoaneurysm formation, arterial thrombosis or arterial dissection.^3^ In addition, common surgical differentials for lower abdominal pain must be considered with this presentation, including bowel obstruction, gastroenteritis, diverticulitis, urinary tract infection, urinary retention and renal colic.

## Investigations/imaging findings

**Figure 2.**
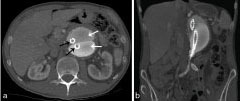
CT images that were mistakenly reported as a likely aortic dissection. (a) Axial image showing dual lumen of the Nellix aortic endograft (black arrows) surrounded and held in position by contrast-injected bags of polymer (white arrows). (b) Coronal image showing the Nellix stent and the surrounding polymer bags.

An urgent CTA of the aorta was requested and reported as suggestive of aortic dissection in light of the impression of a new intimal flap when compared with the pre-operative CTA appearance (Figure 2).

## Treatment

Following the initial CTA report, an urgent “blue light” transfer was arranged to the tertiary referral centre, where the original repair had been carried out, for urgent vascular surgical assessment.

## Outcome

The CT scan was reviewed again at the receiving hospital but it was determined that the appearances were the normal radiographic appearances of a Nellix aortic endoprosthesis. There was no aortic dissection and the patient was treated with laxatives for post-operative constipation and discharged home.

## Discussion

**Figure 3.**
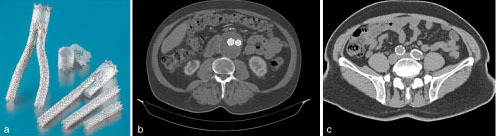
(a) An example of a bifurcated aortic stent graft, demonstrating the modular components (Image courtesy of Dr Donna D'Souza, reproduced from Radiopaedia.org, rID: 36069 published under the Creative Commons Attribution-Noncommercial-Share Alike 3.0 Unported licence). (b) Axial image showing the typical appearance of an endovascular aneurysm repairstent graft within the aneurysm sac (Image courtesy of Dr Sajoscha Sorrentino, reproduced from Radiopaedia.org, rID: 15640 published under the Creative Commons Attribution-Noncommercial-Share Alike 3.0 Unported licence). (c) Axial image showing the typical appearance of an endovascular aneurysm repair stent graft within the common iliac arteries (Image courtesy of RMH Key Conditions, reproduced from Radiopaedia.org, rID: 34236published under the Creative Commons Attribution-Noncommercial-Share Alike 3.0 Unported licence).

EVAR is a minimally invasive technique of aneurysm repair that utilizes stent grafts placed within the aneurysmal sac.^4^ The procedure of EVAR was first described in the early 1990s.^5^ Although the initial techniques utilized a single stent (either aorto-aortic or aorto-uni-iliac), the majority of infrarenal aortic aneurysm repairs are now performed using a bifurcated modular system (Figure 3). Although EVAR has significantly reduced mortality and morbidity compared with an open aneurysm repair,^6^ there are several drawbacks associated with endovascular stent grafts, including proximal and distal graft seal, endoleak, continued aneurysm enlargement and stent kinking, and fracture and migration.

The typical instructions for use of the current devices commonly used to treat an infrarenal aneurysm include the presence of adequate length (≥15 mm) and quality of aneurysmal “neck” proximally (and distally in the iliac arteries), lack of severe angulation/tortuosity and adequate calibre of the access arteries.

Juxtarenal or suprarenal aneurysms pose a greater challenge for endovascular repair. In these instances, a technique called fenestrated EVAR (FEVAR) can be employed, in which the stent grafts are customized with “fenestrations” through which the visceral vessels can be cannulated and then stented.^7^ An alternative to FEVAR for overcoming unfavourable visceral arterial anatomical restrictions is the “chimney grafts”. These grafts have been utilized where FEVAR is inappropriate. Stents are first placed into the aortic visceral branches, and then the main stent graft is deployed with the proximal parts of the graft running parallel to the main aortic endograft, between the wall of the aorta and the main aortic stent.^1^ In this case report, the aneurysm repair involved three "chimney grafts" inserted into the renal arteries and the superior mesenteric artery (Figure 1), in combination with a Nellix aortic endoprosthesis (Figure 2).

The Nellix endoprosthesis was introduced in 2010 to overcome the previously described complications associated with the standard EVAR technique, in particular the issues with stent migration and endoleak.^8^ The key problems with the standard EVAR technique relate to issues with the proximal and distal stent fixation sites; migration (with consequent endoleak) at either end of the stent graft can occur if there is loss of fixation. An additional issue is that a large space can be left between the endograft and the aneurysm wall; retrograde flow from the small branches originating from the aortic sac (*e.g.* lumbar vessels) can continue to pressurize the aneurysmal sac.^6^ A third possibility is that the space left between the graft and the aortic wall could allow lateral movement of the graft, increasing the likelihood of stent migration.

The Nellix endovascular stent is a method of endovascular aneurysm sealing (EVAS) that aims to overcome the pitfalls of standard EVAR by utilizing two “endobags” that are inflated and filled with a fast-curing biostable polymer (a polyethylene glycol-based hydrogel) that surrounds and fixates the bilateral stent lumens to seal the aneurysm sac and stabilize the graft in position (Figure 2).This seals off the space between the stent and the aneurysm wall and prevents retrograde blood flow into the aneurysm sac.

In terms of the evolving radiological appearances of the Nellix graft over time, a recent study was carried out by Karthikesalingam et al^9^ that analysed the post-operative CT images of 68 patients following EVAS. A predictable pattern of evolution was reported, with the polymer-filled endobags initially appearing radiodense with a median radiodensity of 158.3 HU, reducing to a median density of 81.0 HU at 3 months. In addition, the typical sites of endoleak following EVAS were anatomically distinct from those of EVAR, with a Type I endoleak noted at the lateral margins of the aneurysm sac and also in between the two endobags. Another significant and common finding was the presence of gas within the endobags 3 months postoperatively, with complete resolution at 6 months in all cases.

This case report highlights the normal CT appearances of the relatively new Nellix EVAS system that were mistaken as suggestive of an acute aortic dissection (an unusual but described complication) potentially requiring urgent vascular intervention. The radiopaque endobags were incorrectly identified as new false lumens, with the intervening space between the endobags interpreted as a new intimal flap.

## Learning points

1. The Nellix endovascular aneurysm sealing system is a relatively new method of aneurysm repair.
2. The polymer-filled endobags may be contrast filled and the normal radiographic appearances may mimic that of an aortic dissection following a traditional aortic endograft insertion.
3. It is important, where possible, to correlate the details of prosthesis insertion/operative details with radiological appearances.

## Consent

Written informed consent for the case to be published (including images, case history and data) was obtained from the patient for publication of this case report, including accompanying images.
